# Community Social Capital, Family Social Capital, and Self-Rated Health among Older Rural Chinese Adults: Empirical Evidence from Rural Northeastern China

**DOI:** 10.3390/ijerph18115516

**Published:** 2021-05-21

**Authors:** Nan Lu, Shicun Xu, Jingyue Zhang

**Affiliations:** 1 Department of Social Work and Social Policy, School of Sociology and Population Studies, Renmin University of China, Beijing 100872, China; nalv9728@ruc.edu.cn; 2 Sau Po Centre on Ageing, The University of Hong Kong, Hong Kong, China; 3 Department of Population, Resources and Environment, Northeast Asian Studies College, Jilin University, Changchun 130012, China; 4 Northeast Asian Research Center, Jilin University, Changchun 130012, China; 5 Institute of Gender and Culture, Changchun Normal University, Changchun 130032, China; zhangjingyue@ccsfu.edu.cn

**Keywords:** social capital, self-rated health, older adults, rural China

## Abstract

This study investigated the relationships among community social capital, family social capital, and self-rated health of older adults in rural China. Data came from a community survey in Jilin Province, China, in 2019. Using a quota sampling method, 458 respondents aged 60 years or older were recruited. Two-step structural equation modeling was adopted to examine the proposed hypotheses. The relationships between community-based structural social capital, family social capital and self-rated health were statistically significant, whereas the relationship between community-based cognitive social capital and self-rated health was statistically nonsignificant. In order to enhance healthy aging, social capital policies and interventions should be developed to promote not only family social capital indicators (e.g., quality of family relationship and support) but also older adults’ structural social capital indicators (e.g., social participation and volunteering) in rural Chinese contexts.

## 1. Introduction

The proportion of older population has grown rapidly in China. The number of adults aged 65 years old or older reached 180 million in 2020, which represents around 13% of the Chinese population [1]. This process of population aging is expected to continue in the next few decades, especially in terms of oldest-old adults, those aged 80 years or older. In 2050, the Chinese oldest-old adult population will constitute around 25% of the oldest-old adult population in the world [2,3]. Hence, it is crucial to examine modifiable factors of healthy aging, which can provide empirical evidence for the development of aging care policies and interventions.

Self-rated health (SRH) is recognized as an important assessment instrument of healthy aging. SRH is defined as people’s subjective evaluation of their general health status [4]. The literature shows that this subjective indicator is a significant predictor of morbidity and mortality, even when demographic characteristics and chronic diseases were controlled [4,5]. The assessment of SRH is not only easy to implement in research and clinical settings but also has proved to be valid across culture and countries [4,6]. The literature has identified significant factors of SRH, such as age, gender, socioeconomic status, and physical health [4,5,7,8,9]. Furthermore, proponents of the resource hypothesis suggested that people’s subjective assessments of their general health status are affected by not only the severity of illness and family history but also external supportive resources from families and communities [4,10]. In particular, social capital was recognized as a significant modifiable factor of SRH in later life [4,5,11]. However, there is a lack of consensus in terms of the operationalization of social capital, which partially results in inconclusive findings [12,13,14]. Furthermore, although the concept of social capital is contextually dependent, most social capital studies have been conducted in Western contexts. Therefore, the aim of this study was to simultaneously examine the role of family-based and community-based social capital (hereafter, family and community social capital, respectively) in influencing SRH in a rural Chinese context.

### 1.1. Defining Social Capital

The most adopted conceptualization of social capital in the field of health research was proposed by Robert D. Putnam. From a collectivist perspective, Putnam defined social capital as “features of social organization, such as trust, norms, and networks, that can improve the efficiency of society by facilitating coordinated actions” [15]. Social capital can also be defined from an individualist perspective. For example, social capital can be viewed as a form of capital [16], which is based on one’s organization memberships. It is also defined as social resources from social connections with shared social values, reciprocity, norms, and memberships [17]. Instead of focusing on the closure and density of social networks, Lin [18] suggested that bridges in social networks could contribute to not only the diffusion of information, skill, and knowledge, but also social resources from external networks.

For older adults, family and communities are two major sources of social capital [17,19,20,21]. While the present study focused on social resources from older adults’ social connections in their families and communities, social capital was assessed from an individual level which is based on the closure and density of one’s social connections.

Community social capital is multiple dimensional in nature, including a cognitive dimension and a structural dimension [13]. Whereas the former (i.e., cognitive social capital) refers to subjective appraisals (e.g., trust and reciprocity), the latter (i.e., structural social capital) is measured by objective indicators such as organization memberships and social participation [22]. Moreover, family social capital is developed through reciprocity among family members and cultural values and social norms shared in family systems [19]. Family social capital is often assessed by structure of families, frequency of family interaction, and family relationships and support [23].

### 1.2. Family Social Capital and SRH

As previously discussed, social capital should be examined in consideration of surrounding social factors. Given the dominant role of Confucianism in traditional rural Chinese society, adherence to filial piety is important for older rural residents. Confucianism and filial culture have undergone great transitions in China in the past few decades, mainly due to modernization and urbanization. However, older rural residents still prefer their families, especially their adult children, as their main support sources to fulfill their long-term care needs [24,25,26,27].

In the 1950s, urban areas in three northeastern provinces used to be among the most economically developed areas. However, these provinces have been lagged behind both socially and economically compared to the eastern coastal region since the economic reform in 1978. The economic disparities between urban and rural areas widened, rather than reduced, during the economic boom [1]. Rural-to-urban internal migration also became particularly prevalent in northeast China in the past few decades. This means that millions of older rural adults were left behind in rural villages and had relatively lower socioeconomic status as compared to their urban counterparts.

Furthermore, given the nature of China’s two-tier society, rural older adults generally had less access to medical care, pension benefits, education, and employment opportunities in the past few decades [28,29]. Although reforms in health care insurance and pension systems have been initiated in rural regions since the 2000s and achieved relatively high insurance coverage in recent years, the schemes’ benefits tend to be limited among older adults in rural China. The lack of formal long-term care systems further has led to the dominant role of family in the aging care system in rural Chinese communities.

Under such circumstances, family social capital might provide older adults with not only social supportive resources but also a sense of security, life meaning, and fulfillment of filial expectations. Empirical studies have identified a significant relationship between family social capital and SRH [8,30,31]. Given that the network and support component of family social capital (e.g., family relationships and support) was found to have higher impacts on healthy aging indicators than the structural component (e.g., family size) [23,32], family social capital was assessed from the perspective of network and support in this study.

### 1.3. Community Social Capital and SRH

The findings of studies concerning the relationship between community social capital and SRH were mixed. In general, micro- or individual-level social capital indicators have larger effects on SRH than macro- or group-level social capital indicators (e.g., aggregated microlevel social capital measures) [5]. Micro-level cognitive social capital (e.g., trust and reciprocity) has larger effects as compared with structural social capital (e.g., citizenship activities and social participation) in Western and economically developed regions [5,33].

The relationship between community social capital and SRH could vary by culture and socioeconomic status. For example, empirical evidence shows that social trust and reciprocity, which are important indicators of cognitive social capital, are significantly associated with SRH in European countries and Australia [34,35,36]. Social capital was found to be significantly associated with physical and emotional health among older adults in urban China [37]. However, this association was not significant in rural Chinese contexts [37].

Furthermore, although participation in community services and social interactions were found to be significantly associated with SRH in Germany, South Africa, and Australia [35,38,39], other survey and intervention studies showed that some structural social capital indicators such as civic participation were not significant factors of SRH in the contexts of United Kingdom, Spain, and China [36,40,41].

In summary, there are two major research gaps in the literature: first, the majority of social capital research were conducted in Western and developed countries. Given the disparity in socioeconomic status and culture, it is important to conduct social capital research in developing and poor neighborhood settings. Previous research also suggested that social capital could have a higher impact on the well-being of older populations with low socioeconomic status as compared with their richer counterparts [42]. Second, as discussed previously, both community and family are important supportive sources among older rural adults [27]. However, few studies have simultaneously examined the role of family and community social capital in promoting SRH, especially in Eastern Asian and economically underdeveloped contexts. Therefore, empirical evidence from the context of rural China is needed to guide the development of local social capital policy and interventions concerning the promotion of healthy aging among adults who are socially and economically disadvantaged. We proposed the following hypotheses:

1. Family social capital is significantly associated with SRH of older adults in rural China, even after controlling for community social capital.
2. Community-based structural social capital is significantly associated with SRH of older adults in rural China, when family social capital is controlled.
3. Community-based cognitive social capital is significantly associated with SRH of older adults in rural China, when family social capital is controlled.

## 2. Materials and Methods

### 2.1. Sampling

A quota sampling approach was applied to recruit respondents aged 60 years old or older from rural communities in Dongliao county, Jilin province in 2019. As discussed previously, rural-to-urban migration is particularly prevalent in Jilin province. This means millions of adults move from their rural hometowns to urban areas to seek job opportunities, and leave their older family members behind in rural communities. Population aging is also developing rapidly in this region, when the economic development of Jilin province is lagged behind than that in the eastern coastal regions. In specific, Dongliao county features 235 villages and 13 townships, and around one fifth of the local population was aged 60 or older in 2018 (national average level is 17.9%). Therefore, this place is suitable to examine social capital and healthy aging in rural contexts.

The specific sampling procedures were as follows. In the first stage, we randomly selected 16 of the 235 rural villages from Dongliao. In the second stage, 30 respondents were recruited from each of the 16 villages. Given that we did not have access to the complete name lists of local village residents, we cannot randomly select respondents from each village. Instead, we recruited respondents based on referrals from village commissions. In order to enhance the level of sample representativeness, the age and gender ratios of the sample were controlled according to the figures of a locally representative sample based on the latest national consensus. The inclusion criteria were as follows: aged 60 or older, hold a local agricultural hukou status, live in a local village for more than half of the past year, and pass a cognition test [43].

The research team was responsible for conducting standardized interview training six interviewers before data collection. The training topics included the rational of the project, questionnaire design, screening, interview strategies and the coding method. Screening and questionnaires were reviewed by two team leaders on a daily basis during the survey period. All missingness and errors were recorded and corrected. Face-to-face interviews were conducted in local villages (e.g., home and community center). The survey questionnaires gathered information such as the respondents’ physical and mental well-being, social capital, family characteristics, intergenerational exchanges, and socioeconomic status. Ultimately, 482 respondents completed the interview. Response rates were above 90%. We excluded 24 respondents who failed the cognition test. The sample size for data analysis is 458.

### 2.2. Measurements

#### 2.2.1. Outcome Variable

In this study, SRH was assessed by a single question: “How do you feel about your overall health status?” Responses were assessed on a 5-point Likert scale (1 = *very poor*, 2 = *poor*, 3 = *fair*, 4 = *good*, and 5 = *excellent*). The distribution of this variable was positively skewed. Therefore, SRH was recoded as a dichotomous variable (0 = *very poor*, *poor*, *or fair*, 1 = *good or excellent*). This is a commonly adopted approach in previous studies [5].

#### 2.2.2. Social Capital Variable

Family social capital was measured by the Multidimensional Scale of Perceived Social Support [44]. The family-dimension scale has four items. Respondents were asked whether their family members would provide social and emotional support to them when necessary and whether they could discuss important issues with their family members and seek their advice in terms of decision making. Responses were assessed on a 5-point Likert scale (1 = *strongly disagree*, 3 = *neutral*, 5 = *strongly agree*). Average scores were calculated to represent the level of family social capital.

In this study, community social capital is considered as latent variables which cannot be observed directly but can be tested by a range of factor indicators (i.e., observed variables) [45]. Two latent variables were conducted: namely structural social capital and cognitive social capital. Structural social capital was assessed by the following four factor indicators. First, an organization list was provided to the respondents, who reported whether they held memberships in these organizations in the last year. The list included political parties, neighborhood committees, sports groups, religious groups, professional associations, women’s groups, community associations, and charity groups. The responses were measured by a dichotomous variable (0 = *no*, 1 = *yes*). Summed scores were calculated to represent the number of organization memberships. Moreover, the respondents were asked whether they had participated in any activities organized by these organizations (social participation: 1 = *never*, 2 = *one time per year or less*, 3 = *a few times per year*, 4 = *1*–*3 times per month*, 5 = *once per week*, 6 = *twice per week or more*) or collaborated with others to cope with a common problem in the community (citizenship activities: 0 = *no*, 1 = *yes*) in the last year. Regarding volunteering, the respondents were asked whether they engaged in any volunteering activity in the past 30 days (0 = *no*, 1 = *yes*).

Four indicators were used to assess cognitive social capital. The four statements were as follows: (a) “The majority of village residents can be trusted” (i.e., social trust in the rural community); (b) “You consider the local village as a family and yourself as a member of the family” (i.e., a sense of belonging); (c) “Local villagers often help each another” (i.e., perceived helpfulness of other residents); and (d) “Local villagers care about both their interests and others’ benefits” (i.e., willingness to cooperate with other residents). The responses were assessed on a 5-point Likert scale (1 = *strongly disagree*, 3 = *neutral*, 5 = *strongly agree*).

#### 2.2.3. Covariates

The covariates included sociodemographic characteristics, number of children, education level, income, and number of diseases. Age was measured in years. The log value of annual household income was calculated to represent income level. The number of living sons and daughters was calculated to represent the number of children. Gender, marital status, and education levels were measured by dichotomous variables (0 = *male*, 1 = *female*; 0 = *other marital status*, 1 = *married*; 0 = *no formal education*, 1 = *primary school education or higher*). Finally, a list of 14 doctor-diagnosed chronic diseases was shown to the respondents, including cardiovascular diseases, diabetes, hypertension, digestive diseases, and arthritis. They indicated whether they had been diagnosed with each disease, measured dichotomously (0 = *no*, 1 = *yes*). Summed scores were calculated to represent number of diseases.

### 2.3. Data Analysis

We used structural equation modeling (SEM) to build the latent variables of social capital and test the relationship between social capital and SRH [45,46]. SEM is suitable for this research for the following reasons: first, SEM can be used to built latent variables of social capital by estimating different coefficients on different factor loadings (e.g., social trust is treated as a factor indicator of cognitive social capital). Second, one of the methodological merits of SEM is that measurement errors are accounted for in this model. Third, there are a range of fit indexes which can be used to assess the model fit of SEM models. In general, SEM is conducted in two steps. In the first step, we used confirmatory factor analysis to establish a measurement model of social capital. Fit indexes were used to determine whether the model adequately fit the data. The fit indexes and cutoff points were shown below: chi-square test (nonsignificant estimates), Tucker-Lewis index (TLI; estimates more than 0.95), comparative fit index (CFI; estimates more than 0.95), root mean square error of approximation (RMSEA; estimates less than 0.05), and weighted root mean square residual (WRMR; estimates less than 1) [45]. In the second step, a structural model was conducted by entering SRH, family social capital, and covariates. SRH was regressed on family social capital and community social capital (i.e., cognitive and structural social capital), controlling for covariates. Mplus 7.0 was used. 

## 3. Results

### 3.1. Descriptive Statistics

We present the respondents’ sociodemographic characteristics in Table 1. The mean age of older respondents was 69.41 years, and 18.6% were aged 75 or older. Around half were older women, the majority were married, and one third had no formal education. 33% of the respondents’ annual household income was greater than 15,001 RMB. Regarding SRH, 2.8% reported their health status as very poor, 15.3% reported poor SRH, 23.6% reported fair SRH, 49.6% reported good SRH, and 8.5% reported excellent health. Only 10 of 458 respondents had no children (2.2%), and 24.0% of the respondents reported they did not have any of the 14 types of doctor-diagnosed diseases.

### 3.2. Structural Equation Modeling

We established a measurement model of social capital before conducting the structural model. The fit index estimates showed that the model fit was good: χ^2^(65) = 77.185, *p* = 0.1432, RMSEA = 0.020, CFI = 0.993, TLI = 0.991, and WRMR = 0.675. The standardized estimates of factor loadings were from 0.681 to 0.915 for the latent variable of cognitive social capital and from 0.435 to 0.859 for the latent variable of structural social capital.

Family social capital, SRH, and seven covariates were entered in the structural model. The fit index estimates also indicated a good model fit: χ^2^(75) = 93.348, *p* = 0.0743, RMSEA = 0.023, CFI = 0.990, TLI = 0.986, and WRMR = 0.747.

**Figure 1:**
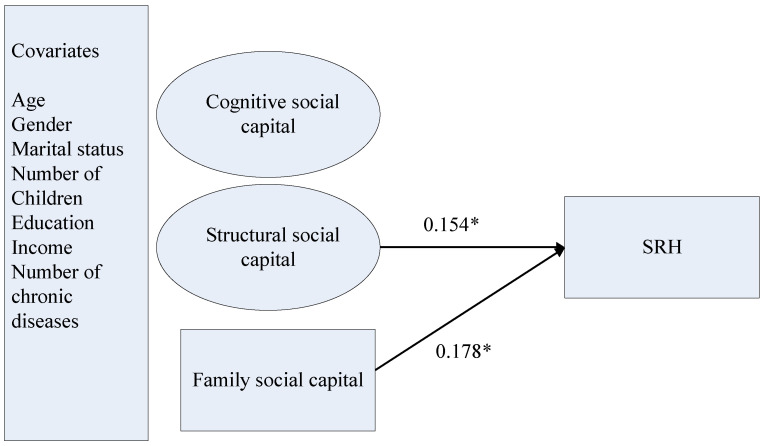
The structural model of family and community social capital, and SRH. Notes: We reported standardized coefficients in this figure. * *p* < 0.05 (two-tailed); SRH = self-rated health; cognitive and structural dimensions of social capital are community-based.

Family social capital was significantly associated with SRH (*b* = 0.371, *SD* = 0.159, *p* < 0.05). The relationship between structural social capital and SRH was also statistically significant (*b* = 0.303, *SD* = 0.119, *p* < 0.05). Although cognitive social capital was significantly associated with SRH when family social capital and structural social capital were not entered in the model (*b* = 0.302, *SD* = 0.143, *p* < 0.05), the relationship was statistically nonsignificant in the final model (*p* > 0.05). Finally, older women were more likely to report poorer SRH than older men (*b* = −0.318, *SD* = 0.136, *p* < 0.05). Respondents with more chronic diseases were more likely to report poorer SRH (*b* = −0.324, *SD* = 0.038, *p* < 0.001). The model is presented in Figure 1.

## 4. Discussion

It is important to accumulate empirical evidence of social capital and SRH in different community contexts. The socioeconomic disparities between rural and urban communities could have accumulating adverse effects on SRH among older rural Chinese adults. Furthermore, given urbanization, modernization, and rural-to-urban migration in the Chinese society, both social and physical environments in urban communities have undergone great transitions. In contrast, there are fewer immigrants in rural communities, and local social norms and cultural values are better preserved in rural regions. This study examines the role of family- and community-based social capital in SRH in older age in rural community contexts in northeastern China. The findings not only support the application of a theoretical framework of family and community social capital in rural Chinese contexts but also provide new evidence to develop social capital intervention programs to enhance SRH among older rural adults.

While social capital is often measured by a single indicator and mainly conducted in urban and developed contexts, we provided a more comprehensive measurement by establishing latent constructs of social capital in rural Chinese contexts. Consistent with previous findings [8,30,31], the results of this study suggest that family social capital is a significant factor for SRH, even when community social capital and covariates such as socioeconomic status and chronic diseases were controlled. Furthermore, previous research based on urban and Western contexts showed that cognitive social capital was a stronger factor of SRH than structural social capital [5,33]. Inconsistent with previous findings [5,33,34,35,36], the findings of this study show that cognitive social capital was not significantly associated with SRH, whereas structural social capital is a stronger factor for SRH as compared with cognitive social capital in rural Chinese contexts. Community social capital could affect SRH through multiple pathways. For example, community social capital could not only promote the diffusion of health-related knowledge and influence health-related behavior but also promote the utilization of home- and community-based services. A sense of belonging and security also could have positive impacts on older adults’ neuroendocrine states, which could further benefit their general health status. For older adults in rural Chinese communities, given that unmet health care needs are a common problem in rural communities with low socioeconomic status [28,29], relying on informal reciprocity among neighbors might not be enough to meet their long-term care needs (e.g., both daily and medical needs). Furthermore, local social services and facilities in the rural communities could be limited. Many older residents might have inadequate health knowledge to care for their personal health due to relatively low educational attainment [21]. For example, under such circumstances, an increase in the frequencies of social participation and volunteering, could promote their health-related behaviors and use of local medical services. These factors could further improve their SRH.

The policy and intervention implications are as follows. First, both community and family social capital can be used in screening tools to identify older populations at risk of poor health in rural Chinese communities, especially older women and those with multiple chronic diseases. Second, social resources from formal support systems does play an important role in promoting SRH in later life, even within considering their inadequate resources and services in rural areas. These formal social resources should be used to improve family relationships and alleviate the care burden on rural families. For example, respite care, caregiver allowances, and financial incentives and tax reductions for family caregivers, could not only alleviate care and financial burdens on rural families, but also help improve the quality of family relationships and intergenerational interactions. Third, social organizations should be developed in rural areas, with the purpose of providing more opportunities for volunteering and citizenship activities. Future social capital interventions should also include educational programs to promote the diffusion of health-related knowledge and services, with an emphasis on common chronic diseases in local communities. Finally, social capital is affected by the neighborhood environment, such as medical services and security in local communities. The development of the physical environment and social services in rural communities could not only have direct positive effects on older adults’ health but also indirectly affect health in later life through promoting social capital by providing additional social resources and opportunities for social interactions. These implications are consistent with the upcoming 14th Five-Year Plan for the Development of China Undertakings for the Aged (the main aging care policy framework in China for 2021–2025) and anti-poverty policy in China.

The present study has some limitations. First, the data were cross-sectional. Therefore, we could not test the direction of causality between social capital and SRH variables. For example, health itself is also recognized as an important resource in later life. Older adults with good health condition could not only be more active in social activities, but also be more optimistic about their lives and tend to be positively assess their social connections in the community. Future longitudinal studies with larger sample sizes and more variables such as optimism and personality traits are needed to address this important issue. Second, we only measured family social capital by using network and support indicators. Future studies are recommended to examine family social capital by more comprehensive measures and further test the interplay between family and community social capital in rural Chinese contexts. Third, we used quota sampling, rather than random sampling to recruit respondents from rural communities in Jilin province. This limits the empirical generalization of the findings. Future studies are needed to further examine SRH trajectory patterns and the role of social capital in influencing the development of SRH trajectories over time.

## 5. Conclusions

The present study simultaneously tested the relationships among community social capital, family social capital, and SRH in older age in rural Chinese community contexts. The results show that the latent variables of social capital were well established based on a rural Chinese sample. Community-based structural social capital and family social capital were two significant factors associated with SRH, whereas community-based cognitive social capital was not significantly associated with SRH in the final model. Future policy and intervention development should not only include community- and family-based social capital in screening tools to identify and provide prevention services to older adults at risk of poor SRH but also develop local social organizations and intervention programs to enhance family relationship and exchanges, social participation, volunteering, and citizenship activities to encourage both personal and collective interests, especially among older women and those with multiple chronic diseases.

## Figures and Tables

**Table 1:** Sample characteristics **(N = 458)**.

	n (%)	Mean (*SD*)
Age		69.41 (6.21)
60–74	373 (81.4)	
75 or above	85 (18.6)	
Gender		
Men	235 (51.3)	
Women	223 (48.7)	
Marrital status		
Married	322 (70.3)	
Other marital status	136 (29.7)	
Education		
No formal education	169 (36.9)	
Primary school education or higher	289 (63.1)	
Annual household income		
RMB15000 or less	306 (66.8)	
RMB15001 or above	152 (33.2)	
SRH		
Very poor/poor/fair	191 (41.7)	
Good/excellent	266 (58.1)	
Number of children		2.42 (1.31)
Number of chronic diseases		1.74 (1.67)

Notes: SRH = self-rated health.

## Data Availability

The data presented in this study are available.
